# The new competitive mechanism of hydrogen bonding interactions and transition process for the hydroxyphenyl imidazo [1, 2-a] pyridine in mixed liquid solution

**DOI:** 10.1038/s41598-017-01780-7

**Published:** 2017-05-08

**Authors:** Yongqing Li, Yunfan Yang, Yong Ding

**Affiliations:** 1 Department of Physics, Liaoning University, Shenyang, 110036 P. R. China; 2 State Key Laboratory of Molecular Reaction Dynamics, Dalian Institute of Chemical Physics, Chinese Academy of Sciences, Dalian, 116023 P. R. China

## Abstract

The new competitive mechanism of intermolecular and intramolecular hydrogen bond can be proposed with an improved mixed model. Upon the photoinduced process, the twisting intramolecular charge transfer (TICT) structure of the hydroxyphenyl imidazo [1, 2-a] pyridine (HPIP) can be obtained. TICT character prompts the fluorescent inactivation via non-radiative decay process. For exploring the photochemical and photophysical properties, the electronic spectra and the infrared (IR) vibrational spectra of titled compounds have been detailedly investigated. In addition, the frontier molecular orbitals (MOs) analysis visually reveals that the unbalanced electron population can give rise to the torsion of molecular structure. To further give an attractive insight into the non-radiative decay process, the potential energy curves have been depicted on the ground state (S_0_), the first excited state (S_1_) and the triple excited state (T_1_). Minimum energy crossing point (MECP) has been found in the S_1_ and T_1_ state. On the MECP, the intersystem crossing (ISC) might be dominant channel. The density functional theory (DFT) and the time-dependent density functional theory (TDDFT) methods have been throughout employed in the S_0_ state, T_1_ state and S_1_ state, respectively. The theoretical results are consistent with experiment in mixed and PCM model.

## Introduction

Hydrogen bond is a non-covalent interaction between electronegative atom Y (acceptor) and covalent bond group X-H (donor)^1^. Generally, the nitrogen, oxygen, or fluorine usually act as electronegative atoms (X, Y). The hydrogen bond X-H···Y plays an important role on the photophysical and photochemical properties, which are correlated with electronegative atoms of hydrogen bond^2^. The human body is comprised of the most abundant several elements including carbon, hydrogen, oxygen and nitrogen, so we will further research the hydrogen bond type that involves the above several elements. To date, an increasing number of researchers have reported that the hydrogen bond plays a critical role in the biochemistry, organic chemistry, photochemistry and physical chemistry, *etc*.^3–7^. Especially the hydrogen bonding interaction has been widely found in the biomolecules such as proteins, nucleic acids, and so on ref. 8. The hydrogen bonding interaction not only be essential to establish stable building blocks of the life, but also act as the sites for the catalyzing reactions of a variety of enzymes. For example, the researchers have reported that the chemical property can be improved *via* changing one single atom in the InsP6 inhibitor, which can strengthen its hydrogen bonding capabilities with toxin molecules. The change has strengthened InsP6 binding to the allosteric modulator by 26-fold^5^. Moreover, hydrogen bond can modulate the metabolism process, such as the hydrogen bond can facilitate the ubiquitous ultra-weak photon emission mode^9, 10^. In addition, the fluorescent phenomena originated from de-excitation process are very widespread in all sorts of scale of biological systems^11^. Therefore, the dynamic mechanism of hydrogen bond will be extensively studied in the photo-excitation process by contemporary investigators^12–14^. Especially, the excited state hydrogen bond strengthening mechanism has been put forward for the first time by Han *et al*.^3, 10, 15–26^. Hydrogen bond strength depends on its bond length, the bond angle, the local dielectric constant, the electronegativity of the donor and acceptor groups, temperature, and pressure, *etc*.^27, 28^. The hydrogen bonding interaction and corresponding dynamical behaviour have been interpreted *via* a variety of photophysical and photochemical phenomena^16, 29–33^. For example, the ESIPT reaction, the intramolecular charge transfer (ICT) and the TICT, *etc*.^19, 28^.

The tautomer structures can be obtained by the ESIPT reaction^28, 34–36^. The ultrafast hydrogen bond strengthening can offer the driving forces for the ESIPT process. Upon the photo-induced process, the electron population has an obvious change for the molecule, which converts from the π character to the π* character. The electron density of proton donor group and acceptor group will reduce and increase, respectively^29^. Therefore, the proton transfer process will be facilitated in the S_1_ state^11, 37^. However, the ESIPT process can be understood as a four-level circular loop model and it is a great important process in the photochemistry, biochemistry and so on refs 38, 39.

As discussed by Toshiki Mutai *et al*. isomer of HPIP originated from ESIPT process had shown an extremely weak fluorescence in the polar solvent tetrahydrofuran (THF). They have drawn a conclusion that the fluorescence yield can be widely enhanced by changing the surroundings from the polar liquid state to the solid state^40^. Actually, the non-radiative decay process plays a prominent role in the biological systems^41, 42^. The long lifetime deactivation process of HPIP will occur *via* the TICT character in the polar solvent, and then the ISC between the S_1_ state and the T_1_ state might be dominant channel for the non-radiative decay. On the MECP, the molecular structures have almost identical energy in the S_1_ and T_1_ state^41^. Subsequently, the radiationless decay will jump from the T_1_ state to the S_0_ state. However, the torsion of HPIP isomer can be prohibited in the solid state surroundings, the nearly co-planar form will emit a drastic fluorescence^40^. In this study, we will carefully investigate the fluorescence quenching mechanism of deactivation process. As Zhao and Liu *et al*. discussed^33, 43^.

In the present work, we utilize a mixed solvent model^44^ to investigate the reaction where a THF molecule bounds to imino group of HPIP, the other solvent can be substituted by polarizable continuum model (PCM). The complex can be established by hydrogen bond interaction. As Fileti *et al*. have discussed that for alcohol-water complexs the stability of complex constitution depends on not only different donor and acceptor molecules, but binding energy of hydrogen bond also is influenced by spatial configurations of complex^45^. Especially, Fileti *et al*.^46^ and Malaspina *et al*.^47^ have accurately provided with the most stable complex of pyridine and water molecule *via* combining Monte Carlo computer simulation and first-principles quantum mechanical calculations method in an aqueous environment^48^. Therefore, in this study HPIP complexs have been certified as most stable structures when considering different spatial configurations of complex. The TICT character can be clearly revealed when we consider the interaction between THF molecule and imine group of HPIP molecule. As Wang *et al*. discussed multiple proton transfer *via* an intermolecular hydrogen-bonded water wire can clearly exhibited the effect of hydrogen bonding dynamics for 3-hydroxypyridine^29^. Otherwise it cannot be primely explained in the conventional PCM model.

## Results and Discussion

**Figure 1:**
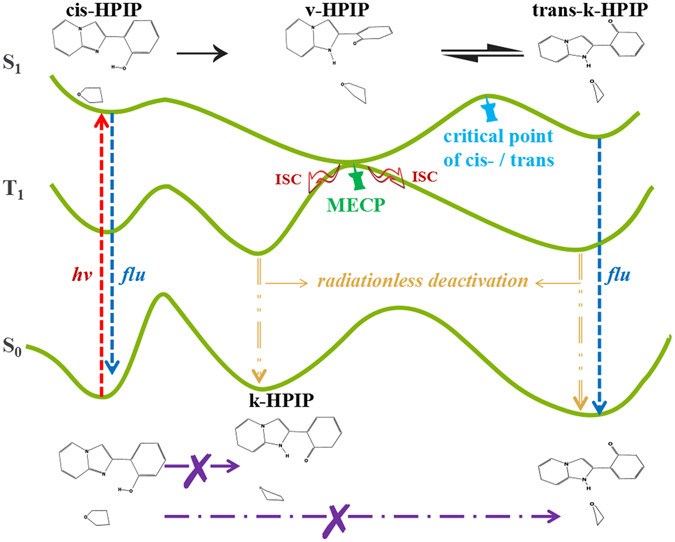
The detailed non-radiative deactivation process.

In this study, we primarily investigate the photophysics and photochemistry properties of HPIP. The HPIP in the THF solvent phase has a dual emitting in the PCM solvation, but the fluorescence of keto form isomer will be totally quenched via the TICT character in the mixed liquid model. However, we are interested in the fluorescence quenching process, and we have speculated that the TICT structure might go through an ISC process from S_1_ state to T_1_ state on the MECP^41, 49–51^. Then, the nonradiative decay will be dominant in the T_1_ → S_0_ state as shown in Fig. 1. Chu *et al*. has introduced the competition mechanism between the intermolecular hydrogen bonding interaction and the ESIPT process^52^. However, in this study a new competitive mechanism of intramolecular and intermolecular hydrogen bond will be investigated in the torsional process.

### Geometric structures and spectra analysis

**Figure 2:**
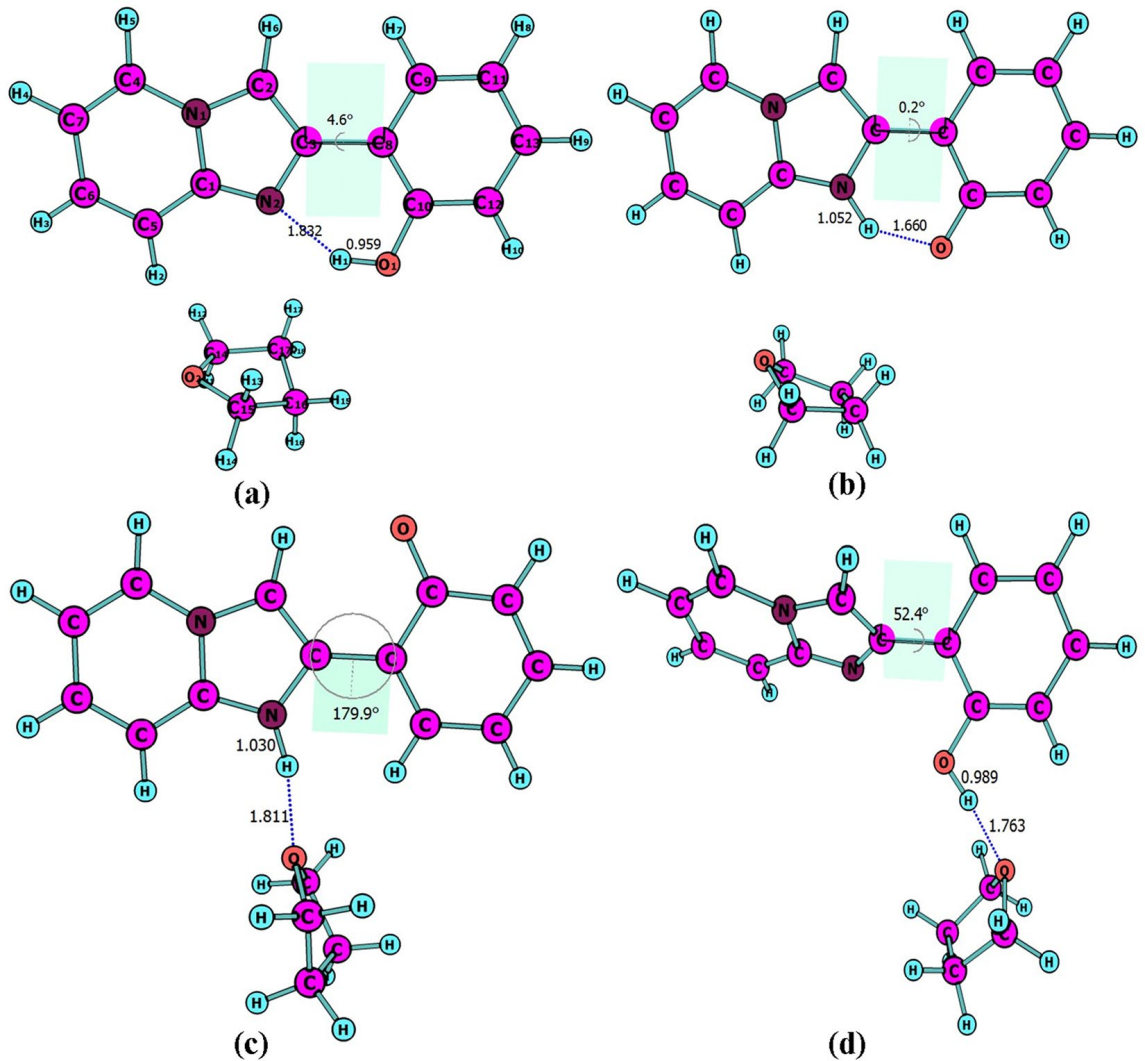
The optimized structures of (**a**) cis-HPIP, (**b**) k-HPIP, (**c**) trans-k-HPIP and (**d**) o-HPIP in the S_0_ state. Some crucial bond parameters have been shown.

**Figure 3:**
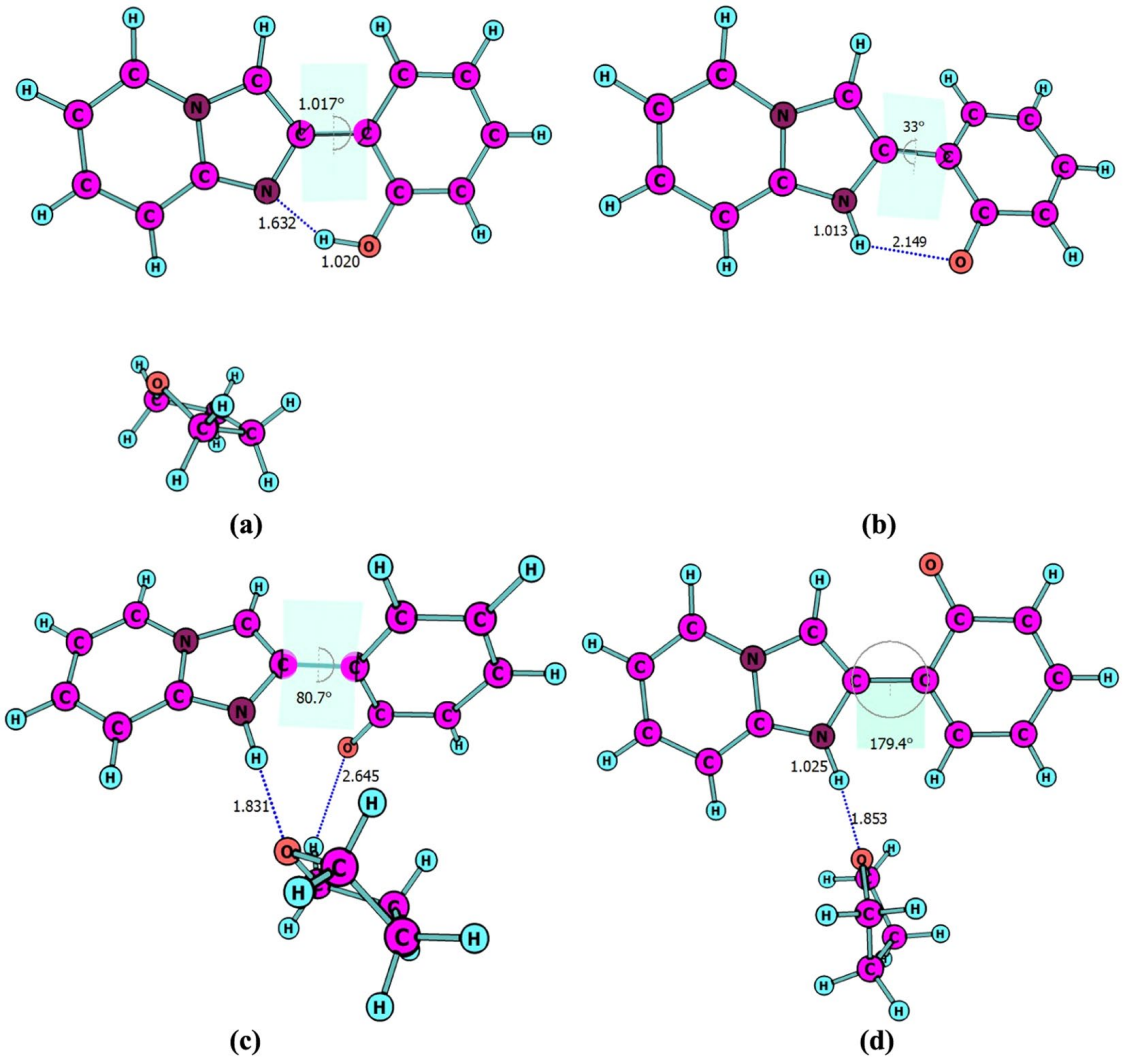
The optimized structures of (**a**) cis-HPIP, (**b**) ik-HPIP, (**c**) v-HPIP, (**d**) trans-k-HPIP in the S_1_ state. Some crucial bond parameters have been shown.

**Figure 4:**
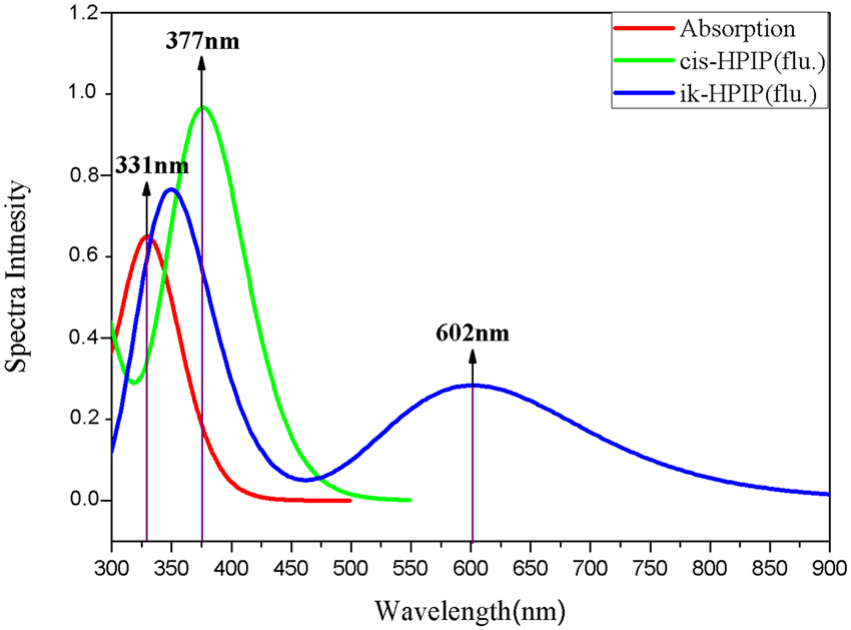
Theoretically simulating the absorption and fluorescence spectra of the HPIP in the PCM solvation, the violet vertical lines stand for the corresponding peak values in the theoretical calculation discussed by Toshiki Mutai *et al*. The detail explanations of curves can be given by the legend on the top right corner.

**Figure 5:**
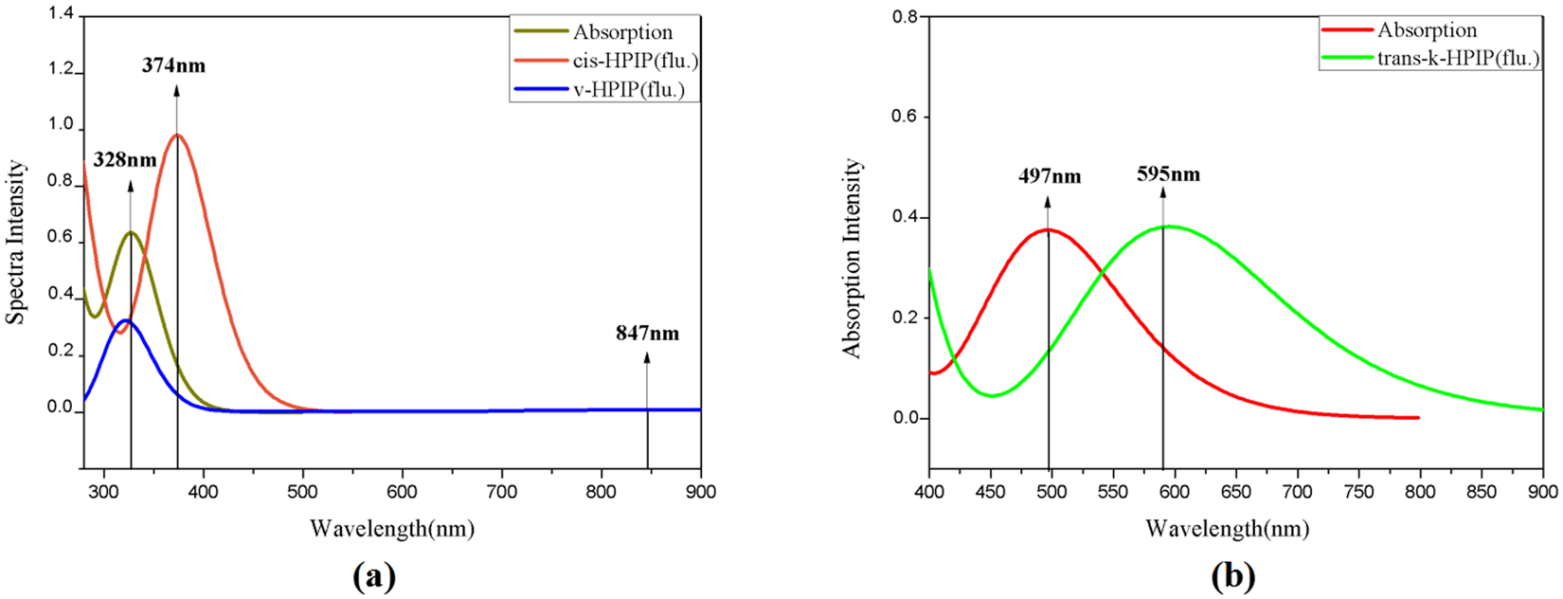
Theoretically simulating the absorption and fluorescence spectra of the HPIP in the mixed liquid model. (**a**) The electron spectra for the cis-HPIP and v-HPIP, (**b**) the electron spectra for the trans-k-HPIP. The detail explanations of curves can be given by the legend on the top right corner.

**Figure 6:**
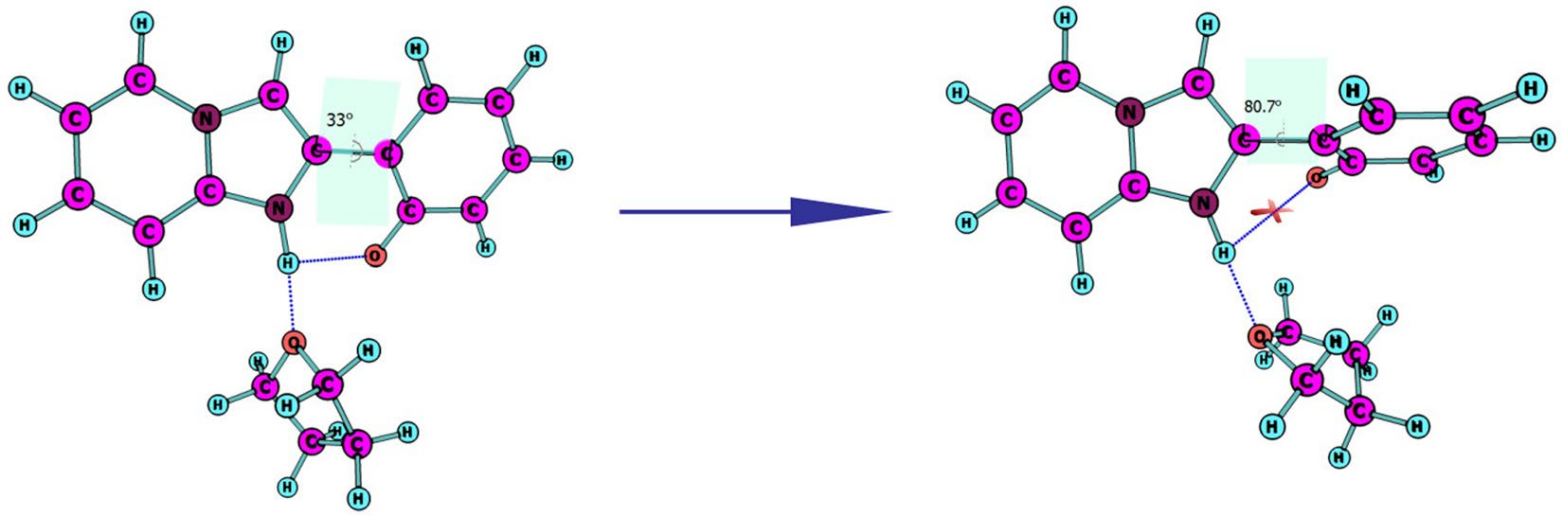
(**a**) the interaction between THF molecule and ik-HPIP leads to (**b**) the formation of v-HPIP in the S_1_ state.

**Figure 7:**
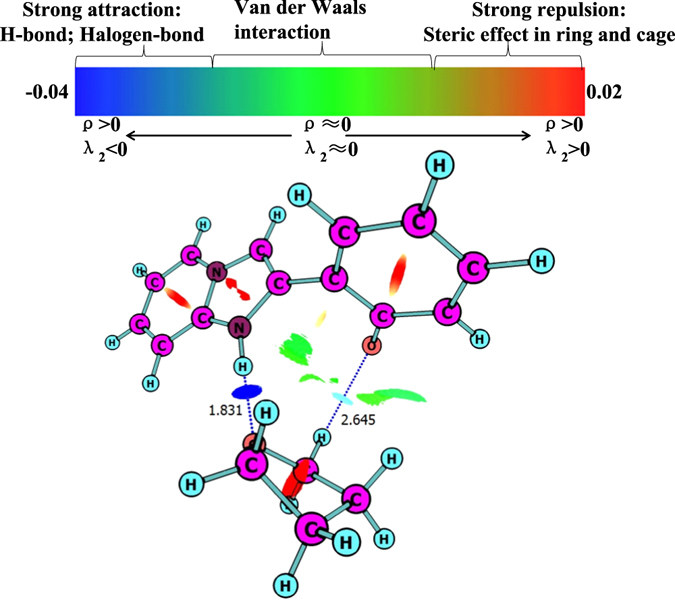
The visual diagram of RDG isosurface and the color gradient axis.

Four stable structures have been found in the S_0_ state, the HPIP (a), the keto form of HPIP (k-HPIP) (b), the trans- keto form HPIP (trans-k-HPIP) (c) and the open-ended intramolecular hydrogen bond HPIP (o-HPIP) (d) have been shown in the Fig. 2. It should be noted that the k-HPIP has not occurred torsion. However, Four stable structures in the S_1_ state, the cis-HPIP (a), in PCM the k-HPIP (ik-HPIP) (b), the nearly vertical isomer form of HPIP (v-HPIP) (c) and the trans-k-HPIP (d) have been shown in the Fig. 3. The k-HPIP form cannot exist in the S_1_ state. After finishing the ESIPT process of HPIP molecule the isomer form has been directly optimized into v-HPIP form. The ik-HPIP and the v-HPIP forms present the rotations to ~33° and ~80° between benzothiazole and phenyl group, respectively. For illustrating the reliability of our computation, the absorption and emission spectra have been calculated in the mixed liquid model. The absorption peak values of the cis-HPIP are located in 331 nm. The emission peak values of the cis-HPIP are located in 377 nm. In addition, the fluorescence peak values of the ik-HPIP are located in 602 nm. The above those peak values have coincided with the computational results of Toshiki Mutai *et al*., which have been shown in Fig. 4. Herein, we have proved the availability of the computing method. The fluorescence of the tautomer form in the S_1_ state exists a Stokes’ shift beyond 200 nm, which indicates that the ik-HPIP form has a drastic change compared with cis-HPIP form. However, the stable structures have not been investigated exclusively in the mixed liquid model. In the Fig. 5, we have calculated the absorption and fluorescence spectra of the cis-HPIP (a) and trans-k-HPIP (b). As shown in Fig. 5(a), the absorption peak values of the cis-HPIP are located in 328 nm. The emission peak values of the cis-HPIP are located in 374 nm. The emission peak value of v-HPIP form is nearly nonluminous located in the about 847 nm. As shown in Fig. 5(b), the absorption peak is about 497 nm and the fluorescence peak is about 595 nm for the trans-k-HPIP. Although the trans-k-HPIP and k-HPIP cannot be directly gained in the S_0_ state, the two structures can be received in the radiationless decay way from the S_1_ state to the S_0_ state. The reaction mechanism has been exhibited in the Fig. 1. Subsequently, for above structures we have dissected carefully the changes of crucial bond parameters in the S_0_ and S_1_ state. As shown in the Tables 1–4, the intriguing bond parameters have been revealed. In Table 1, bond lengths O_1_-H_1_, H_1_···N_2_ of the cis-HPIP are 0.959 Å, 1.832 Å in S_0_ state and are 1.020 Å, 1.632 Å in the S_1_ state, moreover the corresponding bond angle δ(O_1_-H_1_-N_2_) increases from 145.4° to 149.4°. There is a definite conclusion that intramolecular hydrogen bond O_1_-H_1_···N_2_ of the cis-HPIP can be strengthened in the S_1_ state. The dihedral angle δ(N_2_-C_3_C_8_-C_9_) is 4.6° in the S_0_ state, but it reduces to 1.0° in the S_1_ state. The structure of cis-HPIP is near planar in the S_1_ state. The bond parameters of ik-HPIP form has been shown in the Table 2, the bond lengths O_1_···H_1_, H_1_-N_2_ and δ(O_1_-H_1_-N_2_) of ik-HPIP change from 2.150 Å, 1.013 Å and 118.5° to 1.644 Å, 1.055 Å, and 137.9° in the S_1_ → S_0_ state, which indicates that the intramolecular hydrogen bonds O_1_···H_1_-N_2_ of ik-HPIP form is weaker in the S_1_ state than that in the S_0_ state. In addition, we can find that the dihedral angle δ(N_2_-C_3_C_8_-C_9_) of ik-HPIP form decreases from 33° to 0.01° in the S_1_ → S_0_ state from this Table. In the Fig. 2(d), the o-HPIP molecular structure has been optimized with mixed liquid model. We find that the o-HPIP form has the torsion of about 52.4° in the S_0_ state, since the intramolecular hydrogen bond has been destroyed *via* reversing the bond O_1_-H_1_. Therefore, we can conclude that the intramolecular hydrogen bond O_1_-H_1_···N_2_ can preclude the torsion of HPIP structure. In the Table 3, the bond parameters of trans-k-HPIP form have been shown. The dihedral angle δ (N_2_-C_3_C_8_-C_9_) of trans-k-HPIP compared with that of cis-HPIP has twisted about 180°. In addition, a strong intermolecular hydrogen bond N_2_-H_1_···O_2_ has formed. In the S_0_ state, the bond lengths O_1_-H_1_, H_1_-N_2_ and O_2_···H_1_ are 4.954 Å, 1.030 Å and 1.811 Å, respectively. The corresponding bond lengths are 4.945 Å, 1.025 Å and 1.853 Å in the S_1_ state. Finally, the bond parameters of ik-HPIP and v-HPIP form have been shown in the Table 4, it should be noted that the bond length O_1_-H_1_ increases from 2.150 Å to 3.389 Å with torsion of the molecule system, which indicates the torsional behavior has given rise to weakening of the intramolecular hydrogen bond N_2_-H_1_···O_1_. In addition, the intermolecular hydrogen bond N_2_-H_1_···O_2_ has been found in the v-HPIP form, and the bond length O_2_-H_1_ is 1.831 Å. The intermolecular hydrogen bonding interaction can compete with the intramolecular hydrogen bonding interaction of the ik-HPIP form in the mixed liquid model. Yan *et al*. have concluded that the slightly weaker hydrogen bond allows the competition with other type of interaction^53^. Non-coplanar ik-HPIP form results in the intramolecular hydrogen bond is extremely weak. So when the THF molecule approaches to the imino group (=N-H) of ik-HPIP, the intermolecular hydrogen bond will be strengthen and the intramolecular hydrogen bond will be further weaken, the torsion of HPIP molecule can be facilitated as shown in the Fig. 6. We can propose a viewpoint that the intermolecular hydrogen N_2_-H_1_···O_2_ can give rise to the weakening of intramolecular hydrogen bond N_2_-H_1_···O_1_. Furthermore, we have found that a nonatomic ring structure has been generated in the v-HPIP form linked by two intermolecular hydrogen bonds, which are the strong hydrogen bond N_2_-H_1_···O_2_ and the weak hydrogen bond C_14_-H_12_···O_1_. The reduced density gradient (RDG) isosurfaces have been shown in the Fig. 7. Herein, the intensity of hydrogen bond N_2_-H_1_···O_2_ can be visibly compared with that of hydrogen bond C_14_-H_12_···O_1_.

**Table 1:** The bond parameter (bond length (Å), length angle (°) and dihedral angle (°)) of crucial moiety for the cis-HPIP in the S_0_ and S_1_ state.

Parameters of bonds Electronic state	cis-HPIP	
	S_0_	S_1_
O_1_-H_1_	0.959	1.020
H_1_-N_2_	1.832	1.632
δ(O_1_-H_1_-N_2_)	145.4°	149.4°
δ(N_2_-C_3_C_8_-C_9_)	4.6°	1.0°

**Table 2:** The bond parameter (bond length (Å), length angle (°) and dihedral angle (°)) of crucial moiety for the ik-HPIP in the S_0_ and S_1_ state.

Parameters of bonds Electronic state	ik-HPIP	
	S_0_	S_1_
O_1_-H_1_	1.644	2.150
H_1_-N_2_	1.055	1.013
δ(O_1_-H_1_-N_2_)	137.9°	118.5°
δ(N_2_-C_3_C_8_-C_9_)	0.01°	33°

**Table 3:** The bond parameter (bond length (Å), length angle (°) and dihedral angle (°)) of crucial moiety for the trans-k-HPIP in the S_0_ and S_1_ state.

Parameters of bonds Electronic state	trans-k-HPIP	
	S_0_	S_1_
O_1_-H_1_	4.954	4.945
H_1_-N_2_	1.030	1.025
O_2_-H_1_	1.811	1.853
δ(O_1_-H_1_-N_2_)	42.2°	43.9°
δ(O_2_-H_1_-N_2_)	168.0°	174.8°
δ(N_2_-C_3_C_8_-C_9_)	179.9°	179.4°

**Table 4:** The bond parameters (bond length (Å), length angle (°) and dihedral angle (°)) of crucial moiety for the v-HPIP and ik-HPIP in the S_1_ state.

Parameters of bonds Electronic state	v-HPIP	ik-HPIP
	S_1_	S_1_
O_1_-H_1_	3.389	2.150
H_1_-N_2_	1.026	1.013
O_2_-H_1_	1.831	—
H_12_-O_1_	2.645	—
δ(O_1_-H_1_-N_2_)	80.1°	118.5°
δ(O_2_-H_1_-N_2_)	176.5°	—
δ(N_2_-C_3_C_8_-C_9_)	80.7°	33°

**Figure 8:**
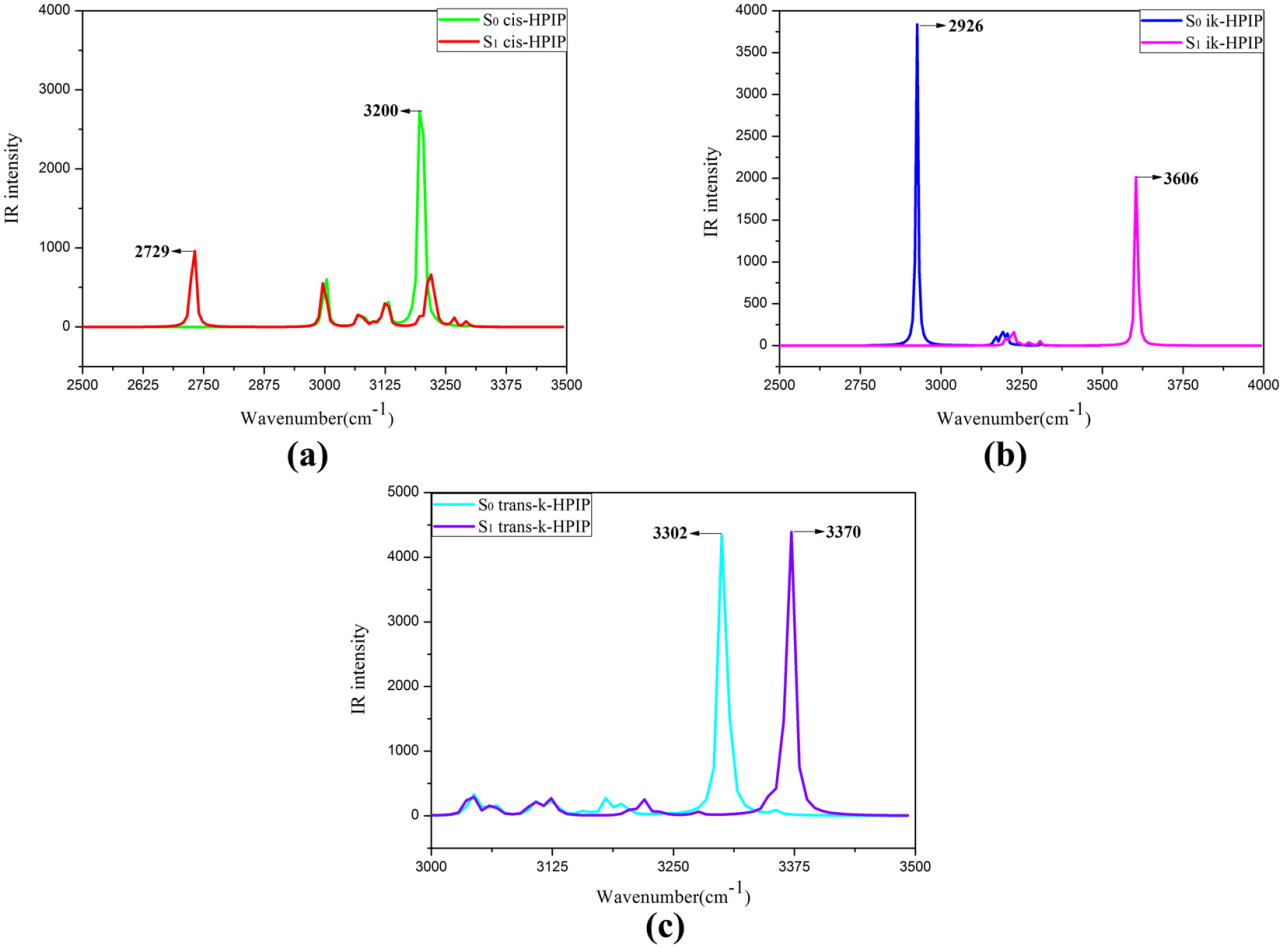
The calculated IR vibrational spectra for (**a**) cis-HPIP in the spectra region of O_1_-H_1_, (**b**) ik-HPIP in the spectra region of N_2_-H_1_, and (**c**) trans-k-HPIP in the spectra region of N_2_-H_1_. The stretching bands of these bonds in the S_0_ and S_1_ state have been shown. The legend can give reader the detail explanations.

For guaranteeing these structures are the true most stable, the corresponding IR vibrational frequency has been calculated. Meanwhile, the anharmonic effects have been considered in stretching frequencies and ΔZPE correction by means of multiplying correction factors 0.991 and 0.977 that Truhlar *et al*. have fitted^54^. The IR vibrational spectra of hydrogen bond have been shown in the Fig. 8. In the Fig. 8(a), the vibrational frequency of O_1_-H_1_ is about 3200 cm^−1^ (the anharmonic frequency is 3171.2 cm^−1^) in the S_0_ state and is about 2729 cm^−1^ (2704.4 cm^−1^) in the S_1_ state for cis-HPIP form. The 471 cm^−1^ (466.8 cm^−1^) red shift indicates that the hydrogen bond O_1_-H_1_···N_2_ is stronger in the S_1_ state. In the Fig. 8(b), the vibrational frequency of the N_2_-H_1_ is tremendously blue-shift 680 cm^−1^ (673.8 cm^−1^) from 2926 cm^−1^ (2899.7 cm^−1^) to 3606 cm^−1^ (3573.5 cm^−1^) in the S_0_ → S_1_ state for the ik-HPIP form, which has indicated that the hydrogen bond N_2_-H_1_···O_1_ is stronger in the S_0_ state. The IR vibrational spectrum of the trans-k-HPIP has been revealed in the Fig. 8(c). The blue-shift 68 cm^−1^ (67.4 cm^−1^) from 3302 cm^−1^ (3272.3 cm^−1^) in the S_0_ state to 3370 cm^−1^ (3339.7 cm^−1^) in the S_1_ state has indicated that the intermolecular hydrogen bond N_2_-H_1_···O_2_ is stronger in the S_0_ state. Upon anharmonic effects the energy of ΔZPE correction for cis-HPIP between the S_0_ and S_1_ state is 0.16 eV within the error range.

### The ESP and the frontier molecular orbitals (MOs) analysis

**Figure 9:**
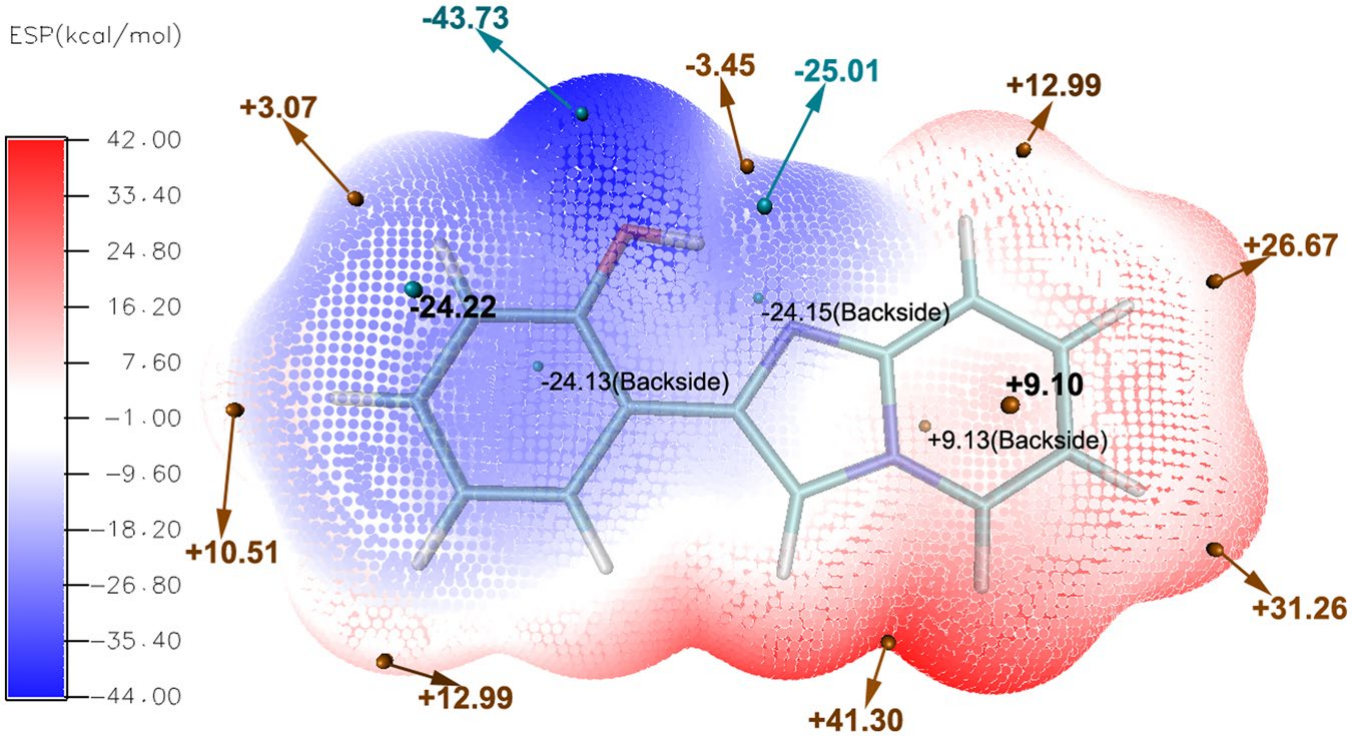
The ESP surface of HPIP molecule srtucure. The corresponding maxima and minima have been labelled in the surface.

**Figure 10:**
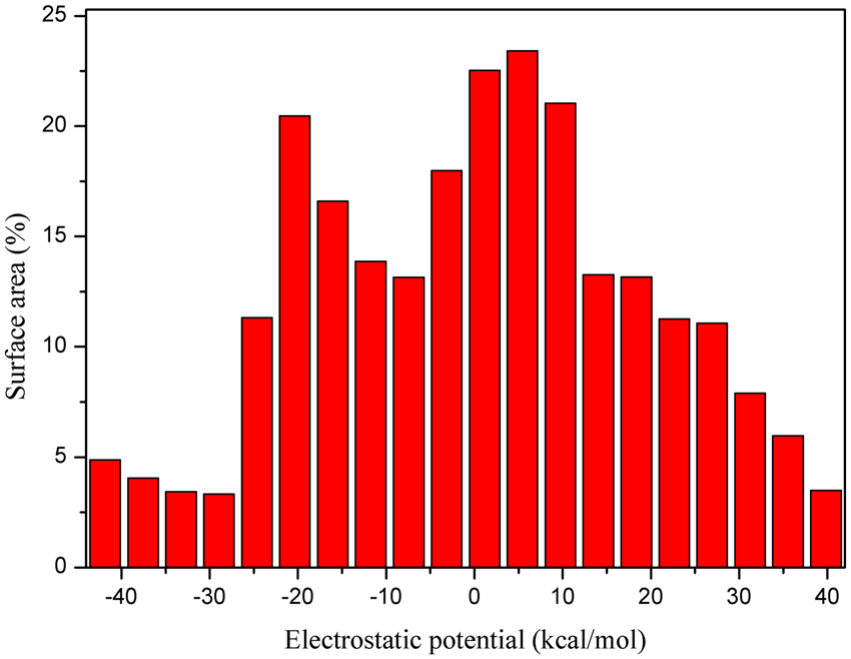
The ESP quantitive distribution column diagram: the X-axis serves as the Surface Area ratio (%), the Y-axis serves as the ESP regions (Kcal/mol).

**Figure 11:**
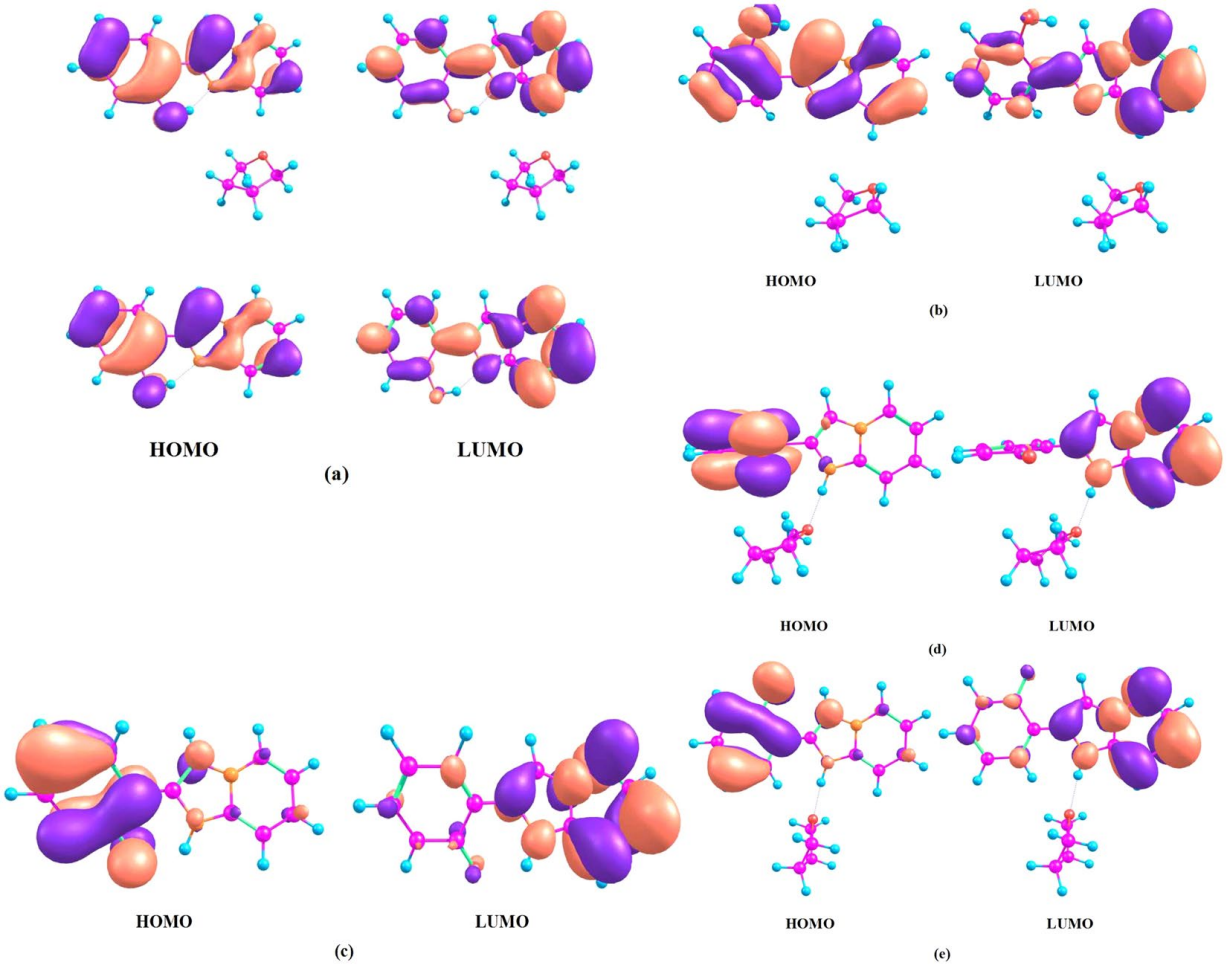
The visual electron population of the frontier molecular orbitals HOMO and LUMO for (**a**) the cis-HPIP in the mixed liquid and PCM solvation, (**b**) np-HPIP, (**c**) ik-HPIP, (**d**) v-HPIP and (**e**) trans-k-HPIP.

We guess the rotation is connected with relative displacement of the electron clouds and the nucleus in molecule under the photo-induced. The centre of gravity of positive and negative charges is tipped, which results in the drastic change of dipole moment. In order to prove our conjectures, the ESP values of HPIP have been calculated by the Multiwfn program^55^ and the ESP surface has been shown in the Fig. 9. The corresponding maxima and minima have been exhibited on the figure, we can clearly found that the negative and positive electrostatic potential exist a drastic polarization distribution on the surface. Moreover, in the Fig. 10, the distribution ratio of the different ESP regions has been quantificationally calculated. The ESP values ranged from −20 Kcal/mol to 20 Kcal/mol widely distribute on the surface area. Thereinto, the negative ESP values mainly origin from the π-electron cloud of aromatic nucleus, the aromatic nucleus of C-H hydrogens mainly contribute to the positive areas. In the Fig. 11, the highest occupied molecular orbital (HOMO) and the lowest unoccupied molecular orbital (LUMO) have been revealed. Upon the photo-induced process, the electron distribution mainly changes from the π character on the HOMO to the π* character on the LUMO. Herein, we only analyze the HOMO and LUMO, because transient excitation mainly stems from the contribution of HOMO → LUMO transition. The transition contribution of ππ* character and corresponding oscillator strength have been listed in the Table 5. In this table, Oscillator strength (f) of ik-HPIP and v-HPIP is 0.1334 and 0.0026, respectively. Their fluorescence compared with the fluorescence of cis-HPIP has been quenched. In the Fig. 11(a), electron redistribution in the PCM is nearly identical to that in the mixed solvation. For further studying the relationship between torsion of molecule structure and electron redistribution, we have obtained non-coplanar HPIP (np-HPIP) form by factitiously twisting the dihedral angle δ(N_2_-C_3_C_8_-C_9_) in the theoretical calculation, in which the np-HPIP molecule cannot be obtained actually in the S_0_ state. The MOs graphs of np-HPIP form have been shown in the Fig. 11(b). Its electron redistribution of the HOMO → LUMO compared with that of cis-HPIP still is not significant change. Therefore, we draw a conclusion that factitious torsion of molecule structure cannot result in the drastic electron redistribution on the np-HPIP. However, the HOMO and LUMO of the ik-HPIP, v-HPIP and trans-k-HPIP have been depicted in the Fig. 11(c,d) and (e). The electron redistribution of these isomeric forms is great different from that of cis-HPIP. It should be noted that electron population of the isomeric forms is unbalanced for HOMO → LUMO. These isomeric forms are zwitterions that the imino group carries positive electric charges and the ketonic oxygen atom carries negative electric charges. ICT character of these zwitterions can lead to the increasing of the dipole moments in the S_1_ state, so the corresponding electron population will be unbalanced in the molecule. Moreover, the unbalanced electron population can further result in the torsion of isomeric forms. It is worth noting that TICT character of the v-HPIP is shown in the Fig. 11(d), the electron population almost entirely transfers from the phenyl group to the benzothiazole group in the HOMO → LUMO.

**Table 5:** The corresponding oscillator strength (f), contribution index (CI) and orbital transition for the different molecular structures in the S_0_ → S_1_ state.

Molecule name	Oscillator strength (f)	CI (%)	Orbital transition
cis-HPIP (PCM)	0.3945	96.27	55 → 56
cis-HPIP (complex)	0.3839	96.08	75 → 76
ik-HPIP	0.1334	99.67	55 → 56
v-HPIP	0.0026	99.69	75 → 76
trans-k-HPIP	0.2318	99.58	75 → 76
np-HPIP	0.2661	94.03	75 → 76

### The analysis of potential energy curves and MECP

**Figure 12:**
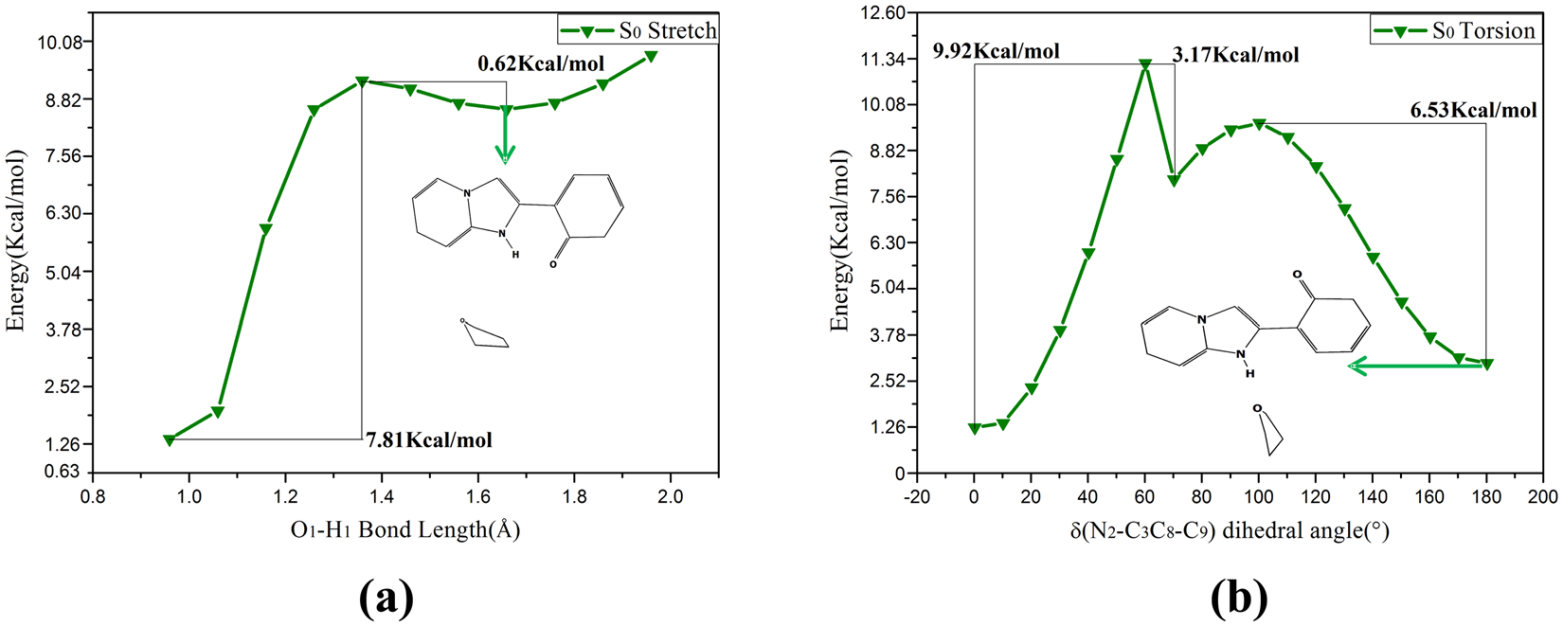
Constructed the potential energy curves of the HPIP: (**a**) the energies of different versus the O_1_-H_1_ bond lengths in the S_0_ state, (**b**) the energies of different structures versus the dihedral angles δ(N_2_-C_3_C_8_-C_9_) in the S_0_ state. The numerical values in the graphs stand for the energy barriers of the reactions.

**Figure 13:**
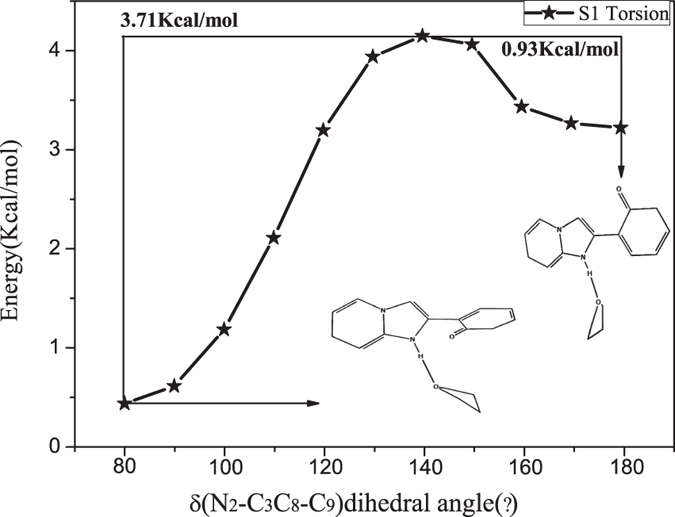
Constructed the potential energy curves of the HPIP: the energies of different structures versus the dihedral angles δ(N_2_-C_3_C_8_-C_9_) in the S_1_ state. The numerical values in the graphs stand for the energy barriers of the reactions.

For further studying the non-radiative decay process, comprehending the optical properties and pathways of electronic transition, the potential energy curves of the HPIP in the S_0_ and S_1_ state have been carefully investigated. The energy of structure is a function of bond length O_1_-H_1_ and dihedral angle δ(N_2_-C_3_C_8_-C_9_), respectively. The potential energy curves will be drawn with the bond length and dihedral angle increased by the fixed step sizes, respectively. The reactive potential barriers and stable structures have been obtained in the potential curves. Firstly, we study the potential energy curves in the S_0_ state. As shown in the Fig. 12, the two potential energy curves are the reactive pathways of (a) intramolecular proton transfer and (b) twisting dihedral angle, respectively. In the Figure the k-HPIP form is a stable structure and it has been used as the original structure in dihedral angle scan. Proton transfer cannot spontaneously occur in the S_0_ state, because the process needs to cross a potential barrier 7.81 Kcal/mol in the Fig. 12(a). Similarly, the dihedral angle torsion cannot also spontaneously occur, and the process needs to cross an energy barrier 9.92 Kcal/mol in the Fig. 12(b). However, the energy instantly reduces about 3.17 Kcal/mol in the torsion process from the Fig. 12(b), because the intermolecular hydrogen bond N_2_-H_1_···O_2_ takes shape in torsion process. Moreover, when the dihedral angle turns to about 180°, the trans-k-HPIP will be a stable structure. Therefore, cis-HPIP, k-HPIP and trans-k-HPIP forms can exist in the S_0_ state, but the energy barriers of the proton transfer and structural torsion process are so high that the two processes cannot spontaneously occur in the S_0_ state. Secondly, we study the potential energy curves in the S_1_ state. In the mixed liquid model the keto form will directly be optimized into the v-HPIP form, so the v-HPIP form has been used as the initial structure in dihedral angle scan. In the Fig. 13 the potential energy curve of twisting dihedral angle has indicated that the v-HPIP form located in about 80° is stable structure, and the stable tran-k-HPIP form located in about 180°. Moreover, the torsion from the v-HPIP to the trans-k-HPIP must get over an energy barrier 3.71 Kcal/mol. The reversed torsion process needs to get over a small energy barrier 0.93 Kcal/mol, which indicates that the trans-k-HPIP can spontaneously revert to v-HPIP form in the S_1_ state. The corresponding potential barrier is the critical point of cis- and trans-isomer.

**Figure 14:**
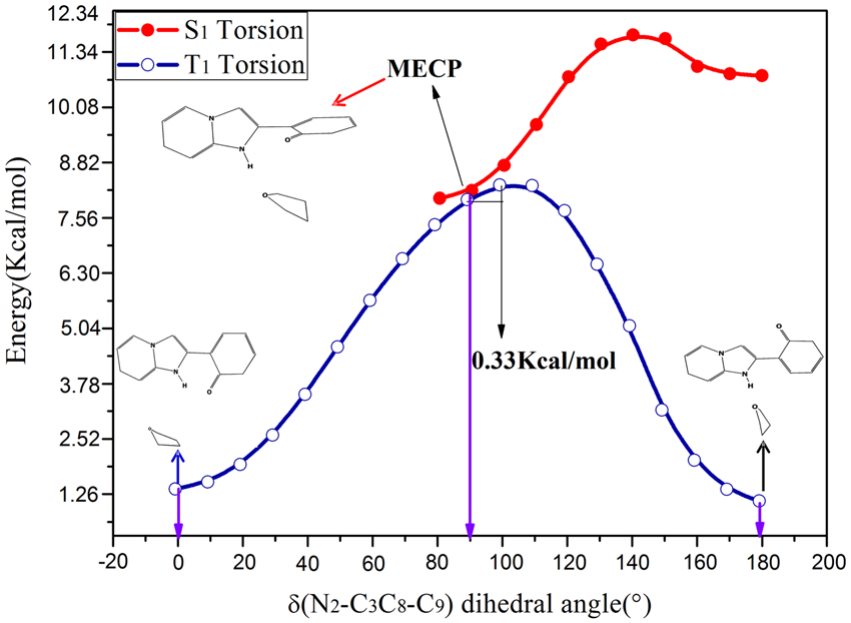
Constructed the potential energy curves of the HPIP: red line: the energies of different structures versus the dihedral angles δ(N_2_-C_3_C_8_-C_9_) in the S_1_ state; blue line: the energies of different structures versus the dihedral angles δ(N_2_-C_3_C_8_-C_9_) in the T_1_ state. The numerical value in the graph stands for the energy barrier of the reaction. The MECP and the corresponding structure have been shown.

To sum up, we have fully explained the reactive pathways for the HPIP complexes *via* analyzing the corresponding potential energy curves in the S_0_ and S_1_ state. However, the non-radiative transition process is still ambiguous, so we have further calculated the potential energy curves of the T_1_ state. Because the oscillator strength of v-HPIP we have calculated is 0.0026, fluorescence of the v-HPIP has been totally quenched. We speculate that the v-HPIP structure might undergo a non-radiative transition from the S_1_ state to the S_0_ state. Therefore, herein searching the MECP has become mainly work for us. As shown in the Fig. 14, the potential energy curves of the torsional dihedral angle in the T_1_ state and S_1_ state have been exhibited. It should be noted that the MECP locates in about 90° in the figure. On this point, the ISC process might be dominant channel in the S_1_ → T_1_ process. In the potential energy curves of the T_1_ state, the MECP structure is extremely unstable, so this structure will fast slide to the k-HPIP form located in about 0° or will get over a negligible energy barrier 0.33 Kcal/mol and then fast slide to the trans-k-HPIP form located in about 180° along with the potential energy curve of T_1_ state. The k-HPIP and trans-k-HPIP existed in the S_0_ state could be obtained from the radiationless decay process. As Harvey *et al*. reported that the reliability of hybrid method has been testified about searching the MECP^56^. Therefore, for prove the reliability of MECP, the sobMECP suite^57^ has been used in this study. It should be noted that the point sobMECP has sought out is about 0.34 Kcal/mol higher than MECP we have confirmed, and their geometries are nearly consistent.

**Figure 15:**
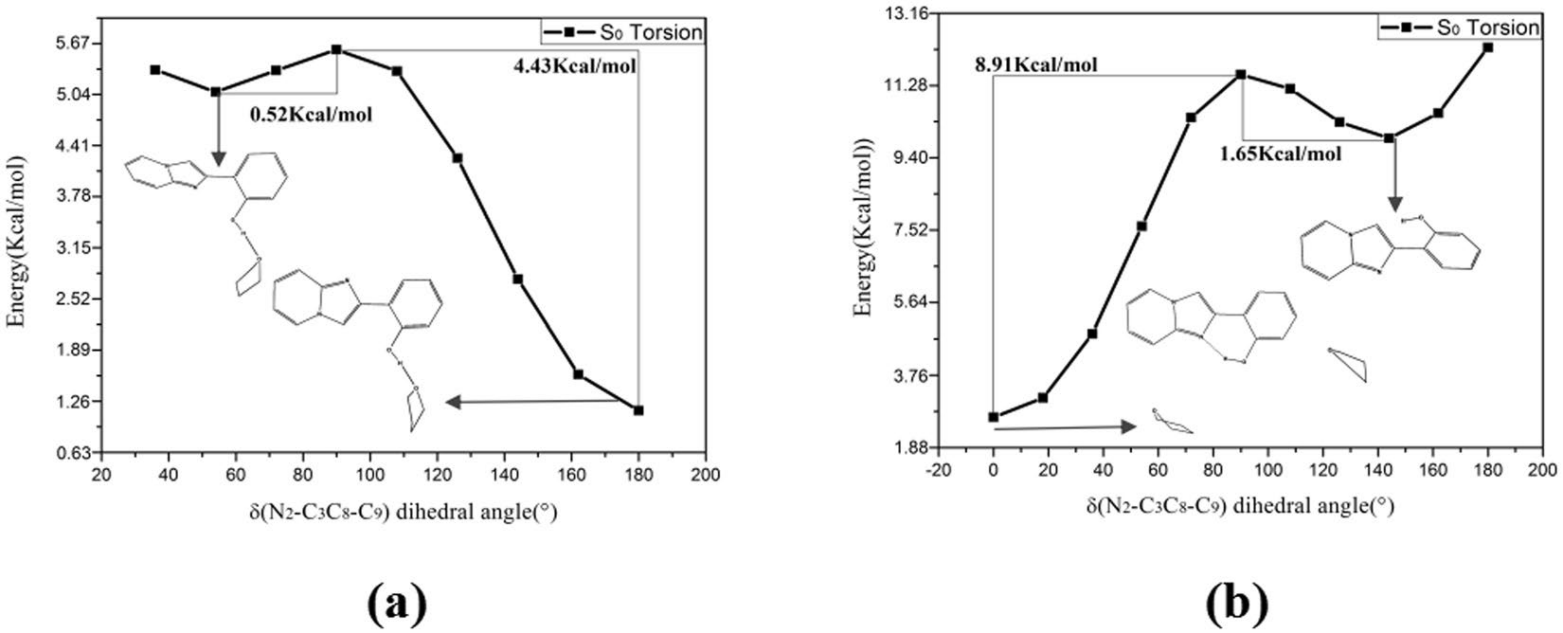
Constructed the potential energy curves of the o-HPIP (**a**) and np-HPIP (**b**): the energies of different structures versus the dihedral angles δ(N_2_-C_3_C_8_-C_9_) in the S_0_ state. The numerical values in the graph stand for the energy barriers of the torsion and reversed torsion process.

In addition, the functions of the potential energy curve versus the dihedral angle δ(N_2_-C_3_C_8_-C_9_) of o-HPIP and np-HPIP forms have been depicted on the Fig. 15. The negligible energy barrier of o-HPIP is 0.52 Kcal/mol and the energy barrier of reversed twisting is 4.43 Kcal/mol in the Fig. 15(a), it is clearly indicated that twisting of o-HPIP can spontaneously occur in the S_0_ state, the energy of o-HPIP form is gradually reduced with the torsional process and the stable structure located in about 180°. On the contrary, in the Fig. 15(b) the energy barrier of np-HPIP is 8.91 Kcal/mol and the energy barrier of reversed twisting is 1.65 Kcal/mol. The stable np-HPIP form locates in about 143.4° and the energy of np-HPIP form is gradually increased with the torsional process. Therefore, the intramolecular hydrogen bond can preclude the torsion of HPIP structure.

## Conclusions

For illustrating the reliability of our computation, we have compared the electronic spectra with that of Toshiki Mutai *et al*. Both of the computed results are tremendous coincidental. The hydrogen bonding strengthening mechanism has been proved *via* analyzing the bond parameters of hydrogen bond in the S_0_ and S_1_ state. In addition, the analysis of IR vibrational spectra can further illustrate the above strengthening mechanism. In this study, competitive mechanism that intermolecular hydrogen bond N_2_-H_1_···O_2_ can weaken interaction of intramolecular hydrogen bond N_2_-H_1_···O_1_ has been primely proved. MOs analysis has indicated that the unbalanced electron population of the keto forms can give rise to the torsion of structures. For further studying the model of non-radiative decay process, comprehending the optical properties and pathways of electronic transition, the potential energy curves have been studied. Herein, we draw a conclusion that the k-HPIP and trans-k-HPIP cannot spontaneously occur in the S_0_ state resulted from calculating the reactive energy barriers. These two structures may be obtained by the non-radiative decay process from S_1_ → T_1_ → S_0_ state. The MECP has further been sought out *via* sobMECP program^57^. Moreover, we have demonstrated that the intramolecular hydrogen bond N_2_-H_1_···O_1_ can preclude the torsion of HPIP form *via* analyzing the o-HPIP form and np-HPIP form and corresponding potential energy curves.

### Computational details

For providing with available information of geometrical configurations, such as the minimum energy, potential energy surface, electron spectra and infrared vibrational spectra, *etc*.^44^. The density functional theory (DFT) and the time-dependent density functional theory (TDDFT) have been throughout employed in the S_0_ state and the S_1_ state, respectively^58–60^. Both of them have been performed at Gaussian 09 program^61^. In this study, we only select to use Becke’s three-parameter hybrid exchange function with the Lee-Yang-Parr gradient-corrected correlation functional (B3LYP)^62–64^, the Pople’s 6–31 G (d) and 6–31 + G (d) triple-ξ quality basis sets are used in this level computation^65^. Herein, the functional B3LYP had been extensively applied in the past few decades, which indicates that B3LYP method is greatly reliable for calculating theoretically in the S_1_ state^15, 16, 30, 31, 66–80^. We have investigated the solvent effect based on the integral equation formalism polarizable continuum model (IEF-PCM)^81–83^. The molecular electrostatic potential (ESP) has been calculated for predicting the nucleophilic and electrophilic sites of target molecule. The ESP surface has been visually portrayed *via* the Visual Molecular Dynamics (VMD) software^84^. and we have also applied the RDG function to investigate the weak interaction types in the Multiwfn program^55^. The visual software Chemcraft has been applied to visualize these figures, such as the molecular structures, the MOs and the RDG^85^. The MECPs were sought out *via* sobMECP, which is a modified version of Harvey’s MECP program^56^ by Tian Lu. The sobMECP is a wrapper of Harvey’s MECP program to simplify the operation of the MECP program^55, 86^.

For performing fully optimized in our molecular system, the hybrid functional method has been carried out in computed point energies and geometries and corresponding gradients. The effective gradients ***f*** and ***g*** have been defined as:

1 $$f=({E}_{1}-{E}_{2})[(\frac{\partial {E}_{1}}{\partial q})-(\frac{\partial {E}_{2}}{\partial q})]=({E}_{1}-{E}_{2}){x}_{1}$$

2 $$g=(\frac{\partial {E}_{1}}{\partial q})-\frac{{x}_{1}}{|{x}_{1}|}[(\frac{\partial {E}_{1}}{\partial q})\cdot \frac{{x}_{1}}{|{x}_{1}|}]$$

where terms *E*_*1*_ and *E*_*2*_ are energies on the two potential energy curves, respectively. The $\partial {E}_{n}/\partial q$ are corresponding partial derivatives of relative nuclear coordinates *q*.

The energy difference *E*_*1*_ − *E*_*2*_ can reduce gradually in the vector ***f*** direction and *E*_*1*_ can reduce in vector ***g*** direction, which the two vectors ***f*** and ***g*** are orthogonal. The MECP can be sought out by optimizing with effective force ***f*** + ***g*** of molecular system. *E*_*1*_ − *E*_*2*_ of two states presented differ spin status can be reduced with descending total energy.

As hybrid method will be used to find MECP, the spin-orbit coupling will not be taken consideration. In two 2*2 Hessian matrices *H*_*1*_ and *H*_*2*_ electronic coupling matrix elements *H*_*12*_ = *H*_*21*_ become zero. Therefore, when the diagonal matrix elements of two matrices are equivalent, the MECP will be a real minimum. However, MECP is not a stable point in 3N-6 dimensions when the hybrid method is taken a consideration. The energy of MECP will be corrected via second-order Taylor expansion for two states:

3 $$\begin{array}{rcl}E & = & {E}_{MECP}+\frac{1}{2}{\rm{\Delta }}{q}^{T}(\frac{|\frac{\partial {E}_{2}}{\partial q}|{H}_{2}}{|{x}_{1}|}-\frac{|\frac{\partial {E}_{2}}{\partial q}|{H}_{1}}{|{x}_{1}|})\\  & = & {E}_{MECP}+\frac{1}{2}{\rm{\Delta }}{q}^{T}{H}_{eff}{\rm{\Delta }}q\end{array}{\rm{\Delta }}q$$

where ∆*q* is the displacement along touching hyperline, which is perpendicular to different gradient *x*_*1*_, the *H*_*eff*_ is diagonal matrix elements of effective Hessian. For nonlinear molecular system, the MECP can be testified as indeed minimum point in 3N-7 dimensions. So this program can approximatively research dynamic measures in nonadiabatic surfaces^56^.
